# MicroRNA-205-5p inhibits three-dimensional spheroid proliferation of ErbB2-overexpressing breast epithelial cells through direct targeting of CLCN3

**DOI:** 10.7717/peerj.7799

**Published:** 2019-10-08

**Authors:** Takayoshi Takeno, Takuya Hasegawa, Hiroki Hasegawa, Yasuyuki Ueno, Ryo Hamataka, Aya Nakajima, Junji Okubo, Koji Sato, Toshiyuki Sakamaki

**Affiliations:** Laboratory of Public Health, Faculty of Pharmacy, Niigata University of Pharmacy and Applied Life Sciences, Niigata, Niigata, Japan

**Keywords:** miR-205-5p, microRNA, CLCN3, Breast cancer, Cell spheroid, ErbB2

## Abstract

We previously reported that microRNA-205-5p (miR-205-5p) is significantly decreased in the ErbB2-overexpressing breast epithelial cell line MCF10A-ErbB2 compared with control cells. In this study, we identified a direct target of miR-205-5p, chloride voltage-gated channel 3 (CLCN3). CLCN3 expression was induced by ErbB2 overexpression; this induced expression was then reduced to control levels by the transfection of the miR-205-5p precursor. In RNA-binding protein immunoprecipitation with Ago1/2/3 antibody, CLCN3 was significantly enriched in 293T embryonic kidney cells with miR-205-5p mimic transfection compared with negative control mimic transfection. In luciferase reporter assays using CLCN3 3′-UTR constructs, the miR-205-5p mimic significantly decreased reporter activity of both wild-type and partial mutant constructs in MCF10A-ErbB2 cells. In contrast, no inhibitory effects of the miR-205-5p mimic were detected using the complete mutant constructs. Since miR-205-5p expression in exosomes derived from MCF10A-neo cells was substantially higher than in exosomes derived from MCF10A-ErbB2 cells, we next investigated whether an exosome-mediated miR-205-5p transfer could control CLCN3 expression. To this end, exosomal miR-205-5p derived from MCF10A-neo cells was functionally transferred to MCF10A-ErbB2 cells, which served to decrease the expression of CLCN3. To assess the roles of CLCN3 in breast cancer, we next performed three-dimensional (3D) spheroid proliferation analyses using MCF10A-ErbB2 cells treated with MCF10A-neo-derived exosomes or CLCN3 shRNA stably expressing SKBR3 and MDA-MB-453 breast cancer cells. Our results showed that both treatment with MCF10A-neo-derived exosome and CLCN3 shRNA expression suppressed 3D spheroid proliferation. Collectively, these novel findings suggest that CLCN3 may be a novel direct target of miR-205-5p and this CLCN3/miR-205-5p interaction may serve a pivotal role in regulating breast cancer cellular proliferation under physiological conditions.

## Introduction

MicroRNAs (miRNAs) are a class of small noncoding RNAs that regulate gene expression post-transcriptionally through binding to the 3′-untranslated regions (3′-UTRs) of target mRNAs. Via this process, miRNAs regulate various cellular activities, including cellular growth, differentiation, development, and apoptosis. Dysregulation of miRNAs is associated with various human diseases, such as cancer (Jiang et al., 2009). Therefore, miRNAs have emerged as promising prognostic and therapeutic tools for cancer management.

A large number of miRNAs have been identified that are differentially expressed during breast cancer progression, and several have been reported to serve as diagnostic and curative targets (Bertoli, Cava & Castiglioni, 2015; Khordadmehr et al., 2019). Among them, microRNA-205-5p (miR-205-5p) is one potential target that negatively correlates with breast cancer invasion, metastasis, and poor prognosis (Markou et al., 2014; Wang et al., 2019a; Xiao et al., 2018). The suspected tumor-suppressive functions of miR-205-5p in breast cancer appear to be due to the direct targeting of several oncogenes, including ERBB3, VEGFA, PKC ε, E2F1, E2F5, ZEB1 and ZEB2 (Greene, Herschkowitz & Rosen, 2010; Gregory et al., 2008; Hashiguchi et al., 2018). Moreover, HMGB3, KLF12, FGF2 and ITGA5 have recently been reported to be direct targets of miR-205-5p in breast cancer (Elgamal et al., 2013; Guan et al., 2016; Hu et al., 2016; Xiao et al., 2018).

Exosomes are secreted extracellular vesicles that carry various intracellular biomolecules, such as proteins, lipids, DNAs, mRNAs, long non-coding RNAs and miRNAs (EL-Andaloussi et al., 2013; Wu et al., 2017). Recent studies have demonstrated that exosomes mediate intercellular communication by transferring the above biomolecules and are involved in the pathogenesis of diseases including cancer (De Toro et al., 2015; Kalluri, 2016; Kucharzewska & Belting, 2013; Valadi et al., 2007). Cancer-derived exosomal miRNAs are becoming of interest for cancer diagnosis and therapy, and exosomal miR-205-5p has been investigated as a potential biomarker in different types of cancer (Aushev et al., 2013; Crentsil, Liu & Sellitti, 2018; Kitdumrongthum et al., 2018; Taylor & Gercel-Taylor, 2008; Wang et al., 2019b).

We previously reported that miR-205-5p is reduced by ErbB2 overexpression and that the ErbB2 tumorigenic capability to proliferate in soft agar is reduced by exogenous transfection of the miR-205-5p precursor (Adachi et al., 2011). In addition, we previously reported that ErbB2 signaling epigenetically suppresses miR-205-5p transcription via the Ras/Raf/MEK/ERK pathway in breast cancer (Hasegawa et al., 2017). Therefore in this study, we further attempted to identify an additional novel target of miR-205-5p in order to understand the comprehensive role of miR-205-5p in breast cancer. To this end, we focused on chloride voltage-gated channel 3 (CLCN3), a member of the voltage-gated chloride channel family. The volume-regulated anion channel (VRAC) contributes to cell volume regulation (Osei-Owusu et al., 2018). Dysfunction of cell volume regulation is one of the characteristics of cancer cells, leading to aberrant cell proliferation and apoptosis (Pedersen, Hoffmann & Novak, 2013). CLCN3 has been reported to play a key role in native VRAC in a variety of cancer cells (Duan, 2011; Habela, Olsen & Sontheimer, 2008; Lemonnier et al., 2004; Mao et al., 2008). Hence, CLCN3 may regulate cell proliferation and apoptosis via a VRAC-related mechanism.

Our findings in this study demonstrated that CLCN3 is a potential direct target of miR-205-5p and regulates 3D spheroid proliferation in ErbB2-overexpressing breast epithelial cells and breast cancer cells.

## Materials & Methods

### Cells

MCF10A-ErbB2 and MCF10A-neo cells were previously generated in our laboratory (Adachi et al., 2011) and cultured in DMEM/F12 with the addition of 5% horse serum, 20 ng/mL EGF, 10 µg/mL insulin, and 500 ng/mL hydrocortisone. The human breast cancer cell lines MDA-MB-453 and SKBR3 cells, as well as the human embryonic kidney cell line 293T, were cultured in DMEM with the addition of 10% fetal bovine serum. DMEM/F12, DMEM, and fetal bovine serum were purchased from Thermo Fisher Scientific (Waltham, MA, USA). EGF, insulin, and hydrocortisone were purchased from Sigma (St. Louis, MO, USA).

### miRNA precursor transfection

The transfection of miRNA Precursors (Pre-miR™ hsa-miR-205-5p miRNA Precursor or Pre-miR™ miRNA Precursor-Negative Control #1, both purchased from Thermo Fisher Scientific) were performed using the RNAiMAX reagent (Thermo Fisher Scientific, Waltham, MA, USA) following the manufacturer’s instruction. Briefly, the cells were seeded in 12-well plates 1 day before transfection, and 15 pmol/well miRNA Precursor was transfected using Lipofectamine RNAiMAX into 30%–50% confluent cells. At 48 h post-transfection, the cells were harvested for RNA extraction. At 72 h post-transfection, the cells were harvested for protein extraction.

### RNA isolation and real-time RT-PCR of CLCN3

Total RNA was purified by RNAiso Plus (Takara Bio, Kusatsu, Shiga, Japan) according to the manufacturer’s instructions and then treated with RNase-free DNase I (Takara Bio). Subsequently, the RNA was cleaned up using an RNeasy Mini kit (Qiagen). Briefly, 2 µg of total RNA was reverse transcribed into cDNA using the High Capacity cDNA Reverse Transcription Kit (Thermo Fisher Scientific). Quantitative real-time PCR was then carried out on an MJ-Mini thermal cycler in conjunction with a MiniOpticon Real-Time PCR system (Bio-Rad, Hercules, CA, USA) under the following conditions: an initial denaturation step at 95 °C for 10 s, followed by 40 cycles at 95 °C for 10 s and 60 °C for 30 s. Dissociation curve analysis was performed for each reaction to guarantee the specificity of amplification. The final concentrations of the PCR reaction components were as follows: 1X SYBR Premix Ex Taq II (Perfect Real Time) (Takara Bio), 0.4 µM forward and reverse primers and 5 µL template cDNA for 20 µL reaction. The primer sequences were as follows: CLCN3 (forward: 5′-ACATGCACCACAACAAAGGC-3′; reverse: 5′-TTTCGGTTTTGAGCCACACG -3′), ZEB2 (forward: 5′-TGTTTCTGCAAGTGCCATCC-3′; reverse: 5′-ACACTGAAGCTGGTGCAAAG-3′) and β-actin (forward: 5′-ATTGCCGACAGGATGCAGA-3′; reverse: 5′-GAGTAC TTGCGCTCAGGAGGA-3′). Expression level of CLCN3 was normalized to β-actin using a standard curve method.

### Real-time RT-PCR of miR-205-5p and miR-200 family members

Ten ng total RNA was reverse transcribed into cDNA using the TaqMan MicroRNA Reverse Transcription Kit (Thermo Fisher Scientific) with the specific primers for hsa-miR-205-5p (Assay ID: 000509), hsa-miR-200a-3p (Assay ID: 000502), hsa-miR-200b-3p (Assay ID: 002251), hsa-miR-200c-3p (Assay ID: 002300), hsa-miR-141-3p (Assay ID: 000463), hsa-miR-429 (Assay ID: 001024), or RNU48 (Assay ID: 001006) included in the TaqMan MicroRNA assay (Thermo Fisher Scientific). Quantitative real-time PCR was then carried out on the same thermal cycler and real-time PCR system as described above under the following conditions: a hot start step at 95 °C for 10 min, followed by 40 cycles at 95 °C for 15 s and 60 °C for 1 min. The final concentrations of the PCR reaction components were as follows: 1X TaqMan Universal PCR Master Mix, No AmpErase UNG (Thermo Fisher Scientific), 1X specific primers for hsa-miR-205-5p, hsa-miR-200a-3p, hsa-miR-200b-3p, hsa-miR-200c-3p, hsa-miR-141-3p, hsa-miR-429 or RNU48 included in TaqMan MicroRNA Assay mix (Thermo Fisher scientific) and 1.33 µL template cDNA for 20 µL reaction. Bio-Rad CFX Manager software was used for data analysis. The relative expression levels of miR-205-5p, hsa-miR-200a-3p, hsa-miR-200b-3p, hsa-miR-200c-3p, hsa-miR-141-3p and hsa-miR-429 were normalized to the endogenous control RNU 48 according to the delta-delta CT method.

### Western blotting

Western blotting was performed as previously described (Adachi et al., 2011). Whole cell lysates were subjected to SDS-PAGE, and separated proteins were transferred to a 0.2-µm PVDF membrane. Blocking was performed with 5% dry milk in 0.05% PBST. The membrane was then blotted with the specific primary antibody. After washing in 0.05% PBST, the membrane was probed with the corresponding secondary antibody conjugated with horseradish peroxidase. After washing in 0.05% PBST, the membrane was visualized by the SuperSignal^®^ West Femto Maximum Sensitivity Substrate (Thermo Fisher Scientific) and analyzed using the ChemiDoc XRS-J image analysis system (Bio-Rad). The antibodies used in this study are shown in the Supplementary Material and Methods.

### RNA-binding protein immunoprecipitation

RNA-binding protein immunoprecipitation (RIP) was performed using the miRNA Target IP kit (Active Motif, Carlsbad, CA, USA) following the manufacturer’s instructions. Briefly, 293T cells (2.5 × 10^6^) were seeded in a 100-mm dish and transfected with 750 pmol of miR-205-5p mimic or negative control mimic using Lipofectamine RNAiMAX. After 24 h, cells were lysed and RIP assay was performed using anti-Ago1/2/3 antibody or negative control IgG. The immunoprecipitated RNA was purified and subjected to real-time RT-PCR analysis. The levels of CLCN3, ZEB2 or β-actin were detected and normalized to the input levels.

### Reporter plasmid construction and site-directed mutagenesis

Template cDNA was synthesized by the RevertAid First Strand cDNA Synthesis Kit (Thermo Fisher Scientific) from Human Mammary Gland total RNA (Takara Bio) according to the manufacturer’s instructions. The 3′-UTR for CLCN3 was PCR amplified from the template cDNA. The following primers were used: CLCN3 3′-UTR (forward: 5′-GGACTAGTGGGTTTTTGCAACATGGTTT-3′; reverse: 5′-TTGAAGCTTGTCTTTGCAATGTTGGAGCA-3′). The PCR amplifications were performed in reaction volumes of 50 µL containing 5 µL 10X Buffer for KOD-Plus Ver.2, 0.3 µM forward and reverse primers, 5 µL 2 mM dNTPs, 3 µL 25 mM MgSO_4,_4% DMSO, 0.02 U/µL KOD-Plus- (TOYOBO, Osaka, Japan), and 1 µL template cDNA using MJ-Mini thermal cycler. The thermal cycling conditions were as follows: 40 cycles of denaturation at 98 °C for 10 s, annealing at 60 °C for 30 s, and extension at 68 °C for 20 s. After digestion with HindIII and SpeI, the final PCR products were inserted into the HindIII/SpeI sites of pMIR-REPORT™ Luciferase (Thermo Fisher Scientific). This construct was named as CLCN3-3′-UTR-wt. Site-directed mutagenesis was then performed using a PrimeSTAR^^®^^ Mutagenesis Basal Kit (Takara Bio) according to the manufacturer’s instructions. The following primers were used: CLCN3-mut1 (forward: 5′-TCCACCTTACGTCCTGTTGTTTGGGGAGGGAAA-3′; reverse: 5′-AGGACGTAAGGTGGAGCATTATTTGCAAACCAT-3′), CLCN3-mut2 (forward: 5′-GAATGGTCCTGTTGTTTGGGGAGGGAAA-3′; reverse: 5′- ACAACAGGACCATTCCACCGCATTAT-3′) and CLCN3-mut3 (forward: 5′- GCTCCACCTTACGAGGAGTTGTTTGGGGAG-3′; reverse: 5′-CTCGTAAGGTGGAGCATTATTTGCAAACCA-3′). The obtained constructs were named as CLCN3-3′-UTR-mut1, CLCN3-3′-UTR-mut2 and CLCN3-3′-UTR-mut3, respectively.

### Reporter assay

Cells were plated in 12-well plates 1 day before transfection and co-transfected with 400 ng/well CLCN3 3′-UTR, CLCN3-3′-UTR-mut1, CLCN3-3′-UTR-mut2 or CLCN3-3′-UTR-mut3, 50 ng/well pGL4.70 *Renilla* luciferase plasmid (Promega), and 45 pmol/well miR-205-5p mirVana™ miRNA mimic or mirVana™ miRNA mimic Negative Control #1 (Thermo Fisher Scientific) by using Lipofectamine 3000 (Thermo Fisher Scientific). At 48 h post-transfection, the cells were lysed in Passive Lysis Buffer (Promega), and *firefly* and *Renilla* luciferase activities were measured using the Dual luciferase reporter assay (Promega) following the manufacturer’s instructions. The relative *firefly* luciferase reporter activities were calculated by normalizing transfection efficiencies according to the *Renilla* luciferase activities.

### Three-dimensional (3D) spheroid proliferation assay

The 3D spheroid proliferation assay was performed using the Cultrex^®^ 3D Spheroid Colorimetric Proliferation/Viability Assay (Trevigen, Gaithersburg, MD) following the manufacturer’s instructions. Briefly, 3,000 cells were plated in 50 µL medium containing Spheroid Formation ECM in a 3D Culture Qualified 96-well Spheroid Formation plate and cultured for 72 h. In an experiment using CLCN3 shRNA stable cells, 50 µL medium was added to each well and cells were cultured for additional 72 h. In an exosome treatment experiment, 50 µL medium plus 10 µL PBS or 10 µL exosomes derived from MCF10A-neo cells were added to each well, and cells were cultured for an additional 72 h. Cellular proliferation was assessed by MTT analysis, and absorbance was measured on a Biotrak II Plate Reader (GE Healthcare, Chicago, IL) at a wavelength of 562 nm, with background subtracted at 690 nm.

### shRNA expression plasmid construction

The retroviral vector pSINsi-DK II-CLCN3 shRNA and the negative control vector pSINsi-DK II-control shRNA were constructed by inserting the pSINsi-DK II Promoter Cassette and the following sense-loop-antisense DNA sequences into Sse8387I and ClaI sites of the pSINsi-DK II vector (Takara Bio): CLCN3 shRNA, DNA-1 sense: 5′-GATCCAAGGCTCATCAAACAGGTAAATAGTGCTCCTGGTTGTTTACCTGTTTGATGAGCCTTTTTTTTAT-3′, DNA-1 antisense: 5′-GTTCCGAGTAGTTTGTCCATTTATCACGAGGACCAACAAATGGACAAACTACTCGGAAAAAAAATAGC-3′; DNA-2 sense: 5′-CTAGAAAGGCTCATCAAACAGGTAAACACAGGGAAGCGAGTCTGTTTACCTGTTTGATGACCTTTTTTTTCCTGCA-3′, DNA-2 antisense: 5′-TTTCCGAGTAGTTTGTCCATTTGTGTCCCTTCGCTCAGACAAATGGACAAACTACTCGAAAAAAAAGG-3′; and control shRNA, DNA-1 sense: 5′-GATCCGTCTTAATCGCGTATAAGGCTAGTGCTCCTGGTTGGCCTTATACGCGATTAAGACTTTTTTAT-3′, DNA-1 antisense: 5′-GCAGAATTAGCGCATATTCCGATCACGAGGACCAACCGGAATATGCGCTAATTCTGAAAAAATAGC-3′; DNA-2 sense: 5′-CTAGAGGCTATTACGACGTTAATCCACAGGGAAGCGAGTCTGGATTAACGTCGTAATAGCCTTTTTTCCTGCA-3′, DNA-2 antisense: 5′-TCCGATAATGCTGCAATTAGGTGTCCCTTCGCTCAGACCTAATTGCAGCATTATCGGAAAAAAGG-3′.

### Stable cell generation

Retroviral infection was performed as previously described (Adachi et al., 2011; Hasegawa et al., 2017). shRNA-expressing retroviruses were prepared by transient co-transfection with pSINsi-DK II-CLCN3 shRNA or pSINsi-DK II-control shRNA and the amphotropic helper virus pSV-A-MLV into 293T cells by using calcium phosphate precipitation. SKBR3 and MDA-MB-453 cells were cultured with fresh retroviral supernatants in the presence of polybrene for 48 h and then subjected to selection by 1.5 mg/mL G418 (Sigma) for SKBR3 and 1 mg/mL G418 for MDA-MB-453.

### Exosome isolation and exosomal RNA purification

Exosomes were isolated using Total Exosome Isolation (from cell culture media) (Thermo Fisher Scientific) following the manufacturer’s instruction. Briefly, 1 × 10^6^ cells were seeded in a 10 cm dish and cultured in serum-containing medium for 24 h. After washing cells with serum-free medium, the cells were cultured in serum-free medium for 48 h. Culture medium was then harvested and centrifuged at 2,000× g for 30 min. The supernatant was incubated with the Total Exosome Isolation (from cell culture media) reagent at 4 °C overnight and then centrifuged at 10,000× g for 1 h at 4 °C. The supernatant was then removed, and the exosome-containing pellet was resuspended in 100 µL PBS. Exosomal RNA was purified using the Total Exosome RNA & Protein Isolation Kit (Thermo Fisher Scientific) following the manufacturer’s instructions. Confirmation of exosome isolation was checked by evaluating exosomal marker protein expression (Fig. S1).

### Exosome treatment

Cells (4 × 10^5^) were seeded in a 6-well plate and cultured in serum-free medium with 60 µL exosome suspension in PBS or 60 µL PBS for 24 h. Cells were harvested and applied to Real-time RT-PCR analysis for miR-205-5p and CLCN3 and 3D spheroid proliferation assays.

## Results

### MiR-205-5p inhibits expression of CLCN3 in breast epithelial cells

**Figure 1:**
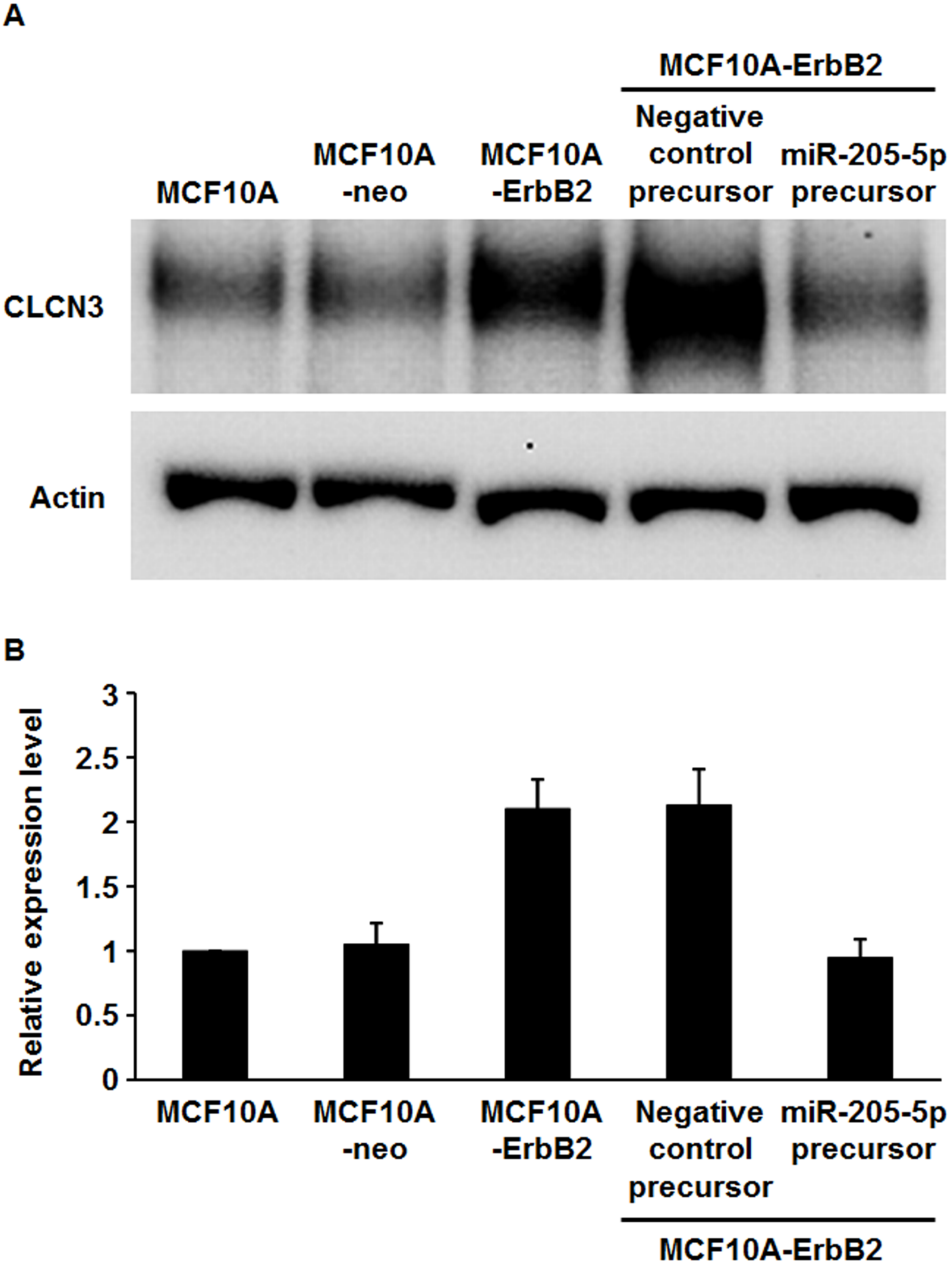
Expression of CLCN3 in ErbB2-overexpressing breast epithelial cells. (A) Western blot analysis of MCF10A, MCF10A-neo, MCF10A-ErbB2 cells and MCF10A-ErbB2 cells transfected with either the miR-205-5p miRNA precursor or negative control precursor. β-actin was used as a control for loading. (B) The graph showed the relative intensities of the bands normalized to β-actin. Data were represented the mean ± SEM of five independent experiments.

We previously established breast epithelial cells that stably overexpress ErbB2 (MCF10A-ErbB2) and the associated control cells (MCF10A-neo). In this previous study, we reported that the overexpression of ErbB2 inhibits the expression of miR-205-5p (Adachi et al., 2011). We next searched for potential target genes of miR-205-5p using *in silico* analysis (miRBLAST-B, Cosmo Bio, Tokyo, Japan) and narrowed down candidate genes by literature search and real-time RT-PCR analysis. Then we selected CLCN3 as one of the candidates. To determine whether miR-205-5p expression correlates with CLCN3 expression in breast epithelial cells, we further examined CLCN3 expression in MCF10A cells, MCF10A-neo cells, MCF10A-ErbB2 cells, negative control precursor-transfected, and miR-205-5p precursor-transfected MCF10A-ErbB2 cells by western blotting. Our results revealed that the expression of CLCN3 increased in MCF10A-ErbB2 cells compared with MCF10A and MCF10A-neo cells and that the elevated CLCN3 expression level was reduced by transfection with the Pre-miR-205-5p precursor (Fig. 1).

**Figure 2:**
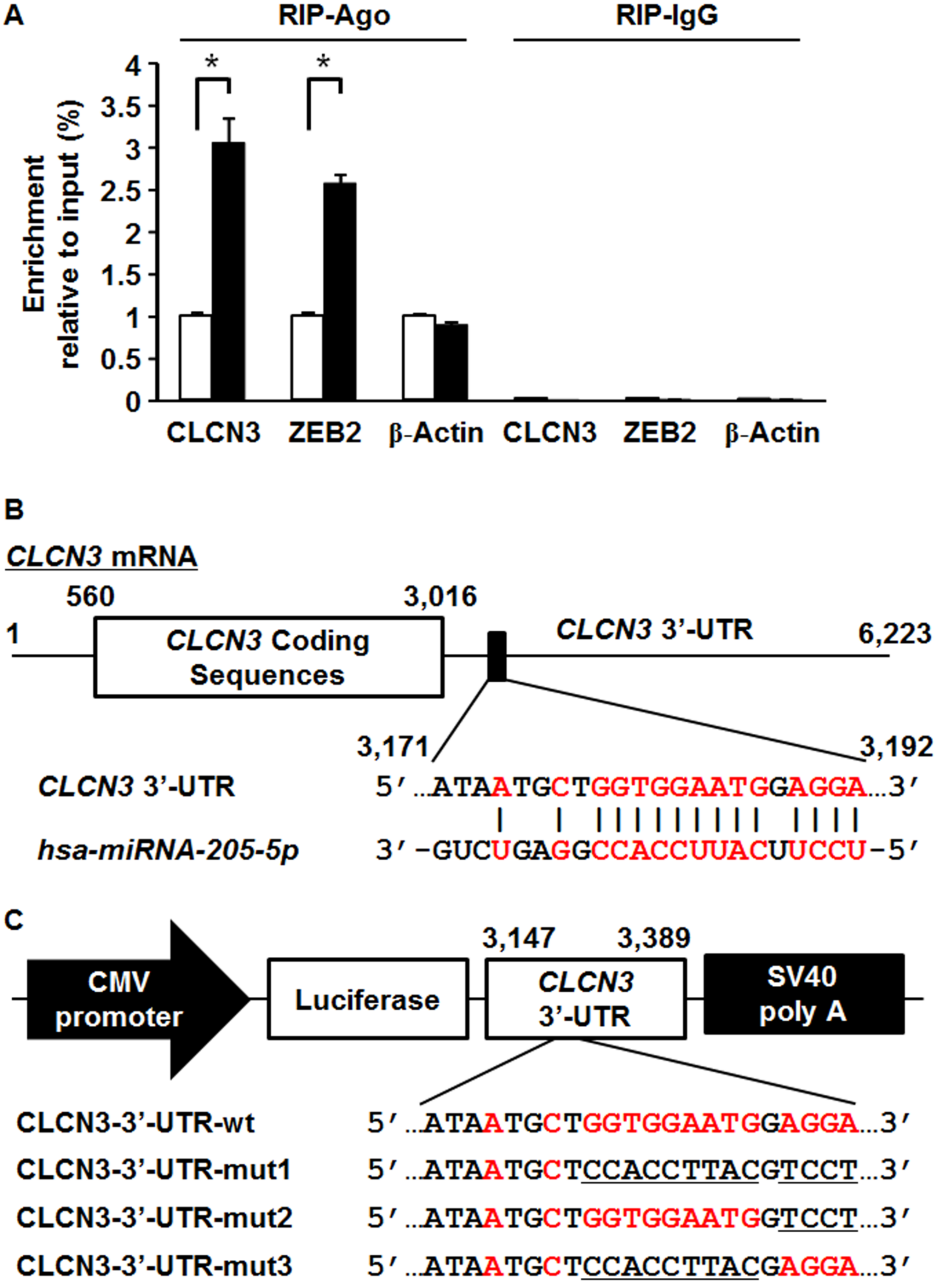
RNA-binding protein immunoprecipitation, predicted miR-205-5p binding site in the CLCN3 3′-UTR and construction of luciferase reporter plasmids. (A) RNA-binding protein immunoprecipitation analysis using a pan-Ago antibody. 293T cells were transfected with miR-205-5p mimic or negative control mimic. At 24 h post-transfection, RIP analysis was performed, and expression of CLCN3, ZEB2 and β-actin was measured by real time RT-PCR. Data were normalized to the input levels and represented as the mean ± SEM of three independent experiments. *p < 0.01 by Student’s *t*-test compared with the negative control mimic. (B) Hsa-miRNA-205-5p/CLCN3 alignment. Predicted miR-205-5p binding site (GenBank Accession No.: NM_001829, 3171–3192) in CLCN3 3′-UTR was indicated. Red letters represent the matched bases. (C) Construction of luciferase reporter plasmids containing wild-type CLCN3 3′-UTR (CLCN3-3t′-UTR-wt) and mutated CLCN3 3′-UTR (CLCN3-3′-UTR-mut1, -mut2, -mut3). Red letters represent the bases matched to CLCN3 3′-UTR. Underlined letters represent the mutated bases. CLCN3-3′-UTR-mut1 corresponds to a complete mutant and CLCN3-3′-UTR-mut2, -mut3 correspond to partial mutants.

**Figure 3:**
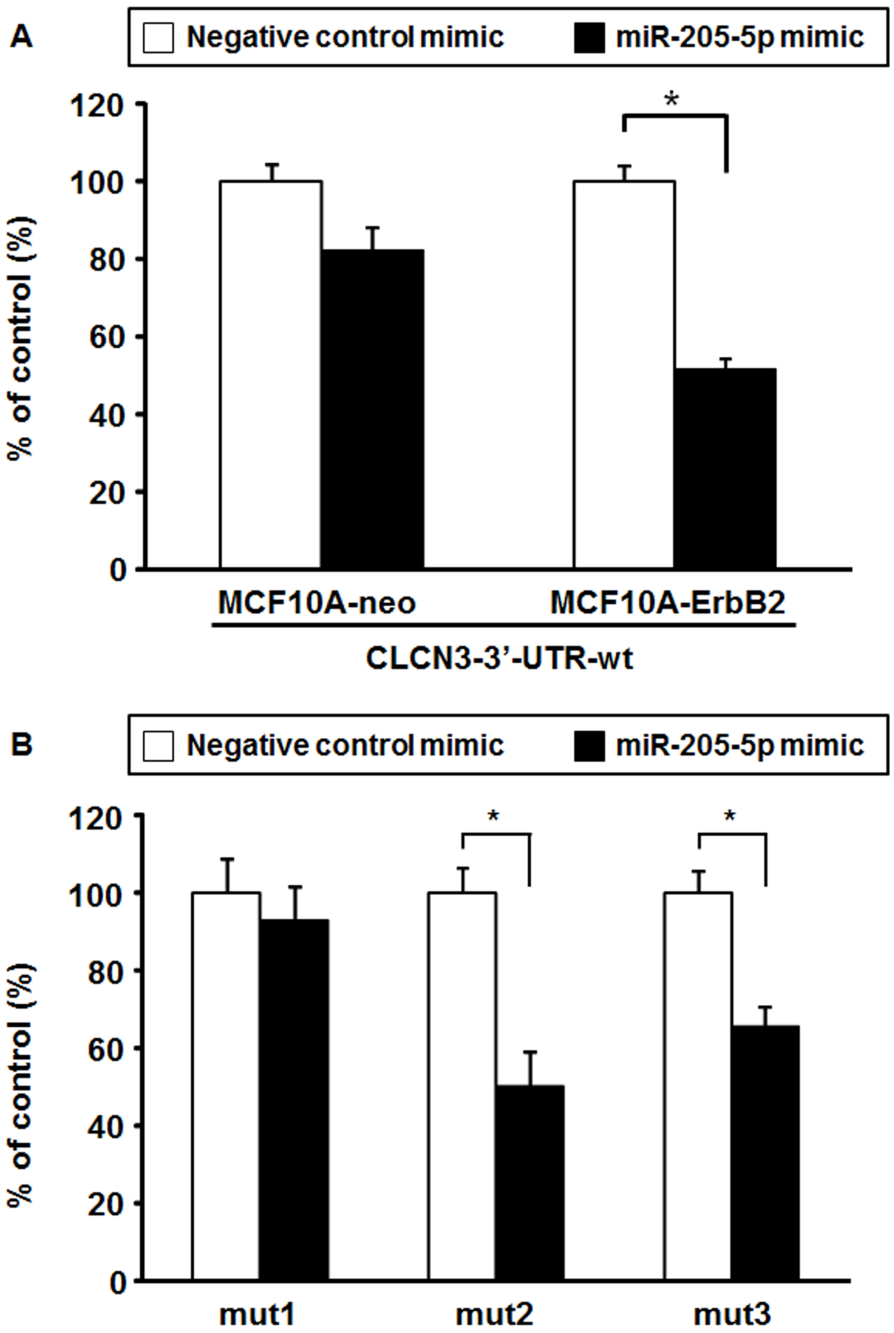
Luciferase reporter assay. (A) Luciferase reporter analysis using the CLCN3 3′-UTR-wt. MCF10A-ErbB2 and MCF10A-neo cells were transiently co-transfected with pGL4.70 *Renilla* luciferase plasmid, CLCN3 3′-UTR-wt and miR-205-5p mimic or negative control mimic. At 48 h post-transfection, luciferase activities were measured. Data were normalized to pGL4.70 *Renilla* luciferase plasmid control and represented as the mean ± SEM of three independent experiments. * *p* < 0.01 by Student’s *t*-test compared with the negative control mimic. (B) Luciferase repoter analysis using the CLCN3-3′-UTR-mut1, -mut2, -mut3. MCF10A-ErbB2 cells were transiently co-transfected with pGL4.70 *Renilla* luciferase plasmid, CLCN3 3′-UTR-mut1, -mut2, -mut3 and miR-205-5p mimic or negative control mimic. At 48 h post-transfection, luciferase activities were measured. Data were normalized to the pGL4.70 *Renilla* luciferase plasmid control and represented as the mean ± SEM of three independent experiments. * *p* < 0.01 by Student’s *t*-test compared with the negative control mimic.

### MiR-205-5p directly targets CLCN3 3′-UTR in breast epithelial cells

An Argonaute protein (Ago) plays a crucial role in the maturation process of miRNAs as a component of the RNA-induced silencing complex. We next performed RIP assay with anti-Ago1/2/3 antibody to validate the interaction between miR-205-5p and CLCN3. RIP assay revealed that the relative enrichment of CLCN3 in Ago immunoprecipitation complex was significantly increased in 293T cells transfected with miR-205-5p mimic compared with negative control mimic group (Fig. 2A). MiR-205-5p mimic transfection resulted in the similar enrichment of ZEB2, a known target of miR-205-5p, whereas didn’t change the enrichment level of β-actin. We further evaluated whether CLCN3 is a direct target of miR-205-5p. We predicted a putative miR-205-5p binding site in the CLCN3 3′-UTR (Fig. 2B) and constructed luciferase reporter plasmids containing wild-type CLCN3 3′-UTR or the three different mutations at the putative miR-205-5p binding site (Fig. 2C). To this end, we transfected the reporter plasmids with either the miR-205-5p mimic or the negative control mimic into MCF10A-ErbB2 or MCF10A-neo cells to determine the reporter activities. Our results indicated that the reporter activity of CLCN3-3′-UTR-wt was significantly decreased by the miR-205-5p mimic co-transfection in MCF10A-ErbB2 cells (Fig. 3A). Moreover, the reporter activities of the partial mutants, CLCN3-3′-UTR-mut2 and CLCN3-3′-UTR-mut3, were significantly decreased by miR-205-5p mimic co-transfection in MCF10A-ErbB2 cells, whereas the reporter activity of the complete mutant, CLCN3-3′-UTR-mut1, was not significantly decreased (Fig. 3B).

**Figure 4:**
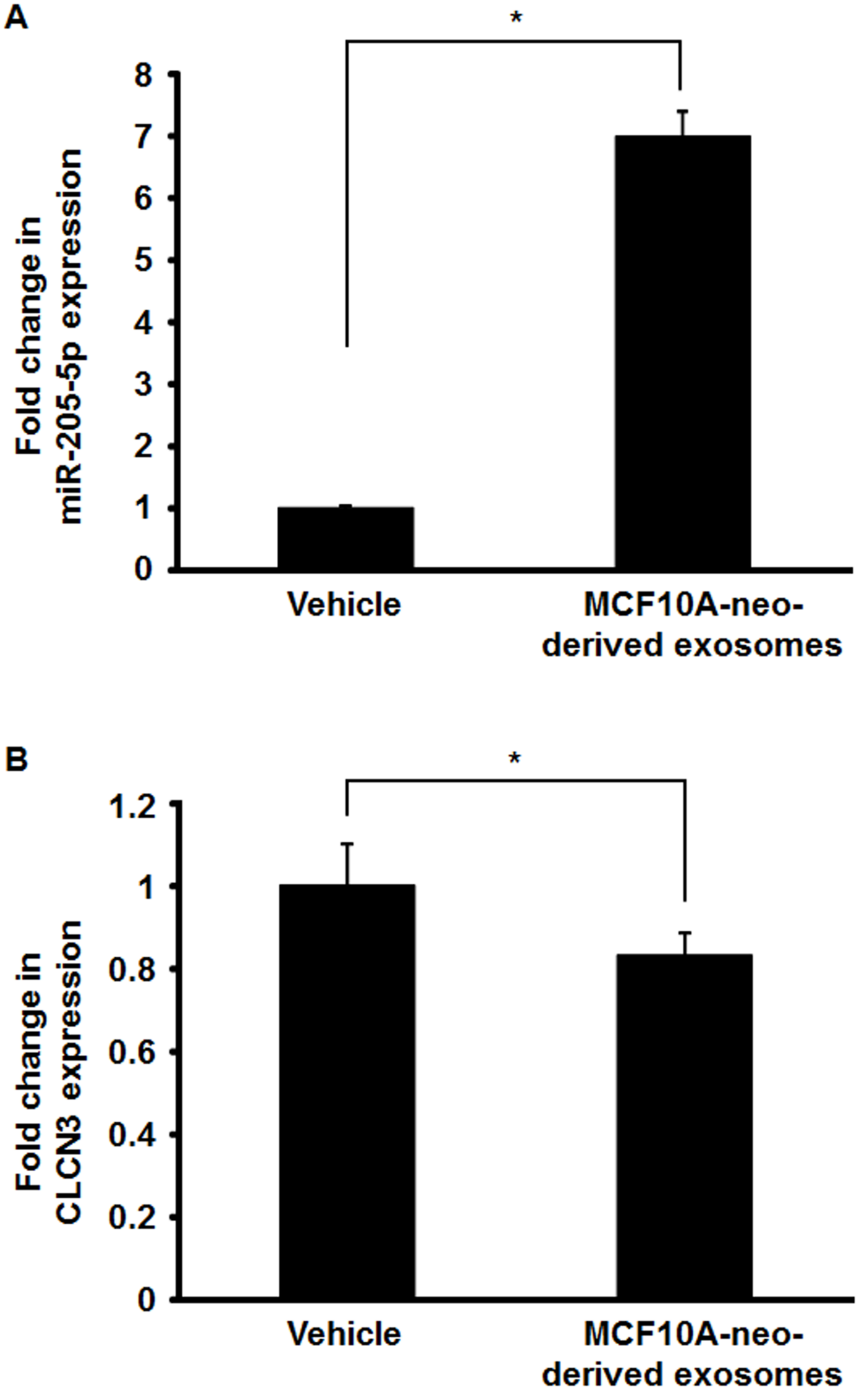
Effect of the treatment with MCF10A-neo-derived exosome on the expression of miR-205-5p and CLCN3 in MCF10A-ErbB2 cells. (A) Real-time RT-PCR analysis for miR-205-5p. MCF10A-ErbB2 cells were treated with MCF10A-neo-derived exosome for 24 h. Data were normalized to vehicle control and represented as the mean ± SEM of three independent experiments. *p < 0.01 by Student’s *t*-test compared with vehicle. (B) Real-time RT-PCR analysis for CLCN3. MCF10A-ErbB2 cells were treated with MCF10A-neo-derived exosome for 24 h. Data were normalized to vehicle control and represented as the mean ± SEM of three independent experiments. *p < 0.05 by Student’s *t*-test compared with vehicle.

### Functional exosomal miRNA is transferred into ErbB2-overexpressing breast epithelial cells

Since the expression of miR-205-5p was significantly reduced in MCF10A-ErbB2 cells compared with MCF10A-neo cells as previously described (Adachi et al., 2011), we next determined the expression of miR-205-5p in the exosomes from these cells. We found that miR-205-5p expression in exosomes isolated from MCF10-neo cells was much higher than in exosomes isolated from MCF10-ErbB2 cells (Fig. S2). It has been reported that exosomal miRNAs can be transferred between cells and mediate target gene repression and physiological function (Bovy et al., 2015; Mittelbrunn et al., 2011; Santos et al., 2016). Therefore, we treated MCF10A-ErbB2 cells with exosomes derived from MCF10A-neo cells to determine whether functional miR-205-5p could be transferred. Our results showed that miR-205-5p expression in MCF10A-ErbB2 cells treated with MCF10A-neo-derived exosomes was increased by about 7-fold compared with vehicle (PBS) treatment (Fig. 4A). Moreover, CLCN3 expression in MCF10A-ErbB2 cells treated with MCF10A-neo-derived exosomes was significantly decreased compared with vehicle treatment (Fig. 4B).

### CLCN3 mediates 3D spheroid proliferation in ErbB2-overexpressing breast epithelial cells and breast cancer cells

**Figure 5:**
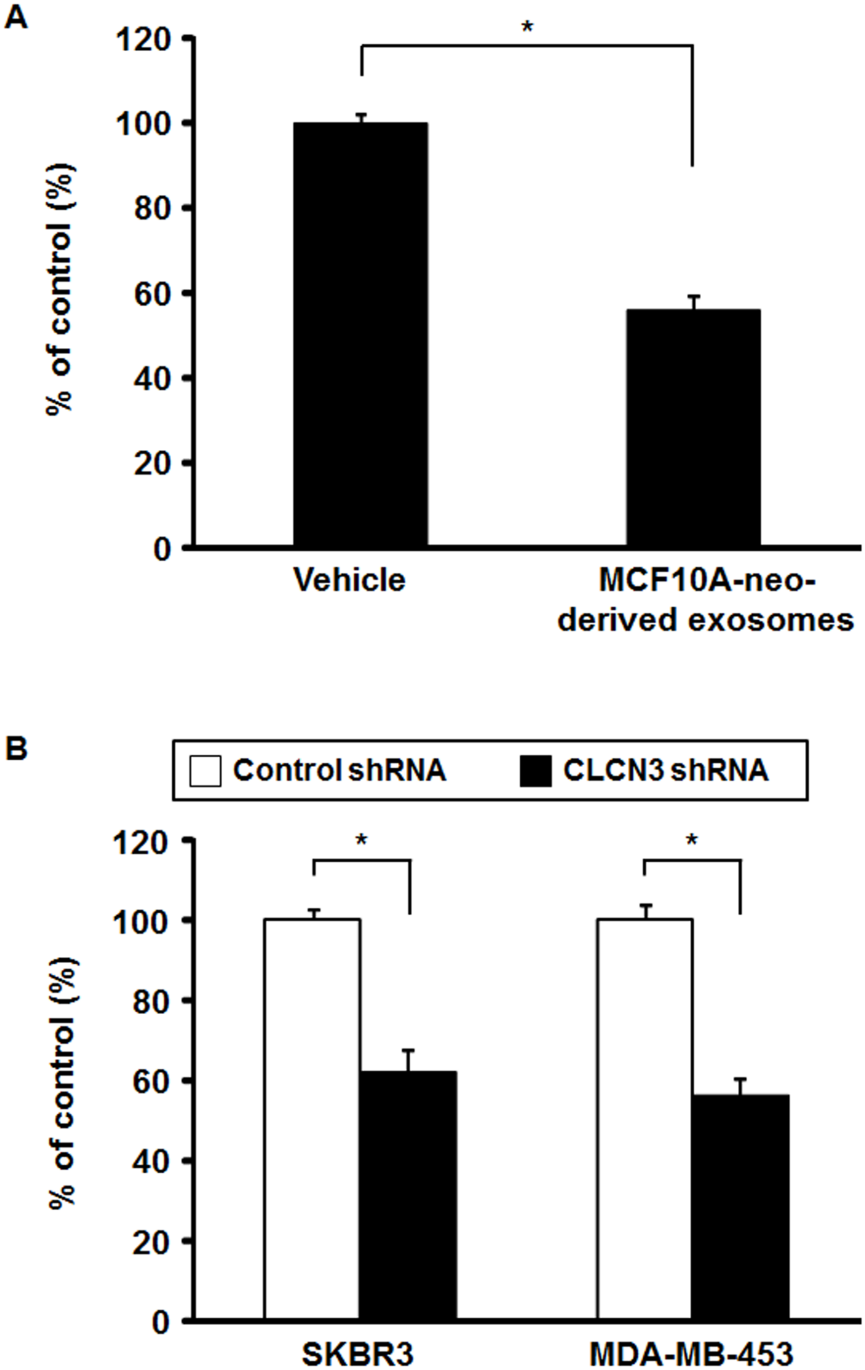
3D spheroid proliferation assay. (A) MCF10A-ErbB2 cells were pre-treated with MCF10A-neo-derived exosome for 24 h and applied for 3D spheroid proliferation assay for 6 days. Data were normalized to vehicle control and represented as the mean ± SEM of three independent experiments. *p < 0.01 by Student’s *t*-test compared with vehicle. (B) CLCN3 shRNA or control shRNA stably expressing SKBR3 and MDA-MB-453 cells were applied for 3D spheroid proliferation assay for 6 days. MTT analysis was performed. Data were normalized to control shRNA stable cells and represented as the mean ± SEM of three independent experiments. *p < 0.01 by Student’s *t*-test compared with control shRNA stable cells.

Since our data indicated that CLCN3 is one of the potential targets of miR-205-5p, we investigated the possible biological function of CLCN3 in ErbB2-overexpressing breast epithelial cells and breast cancer cells. We analyzed 3D spheroid proliferation of MCF10A-ErbB2 cells treated with exosomes derived from MCF10A-neo cells because we previously found that miR-205-5p inhibited 3D colony formation in soft agar using MCF10A-ErbB2 cells. Our results showed that the treatment of MCF10A-neo-derived exosomes decreased 3D spheroid proliferation by about 40% compared with vehicle (Fig. 5A). In addition, we established CLCN3 shRNA or control shRNA stably expressing cells using the ErbB2-overexpressing breast cancer cell lines SKBR3 and MDA-MB-453 (Fig. S3) and analyzed the 3D spheroid proliferation of these stable cells. Inhibition of CLCN3 expression in 3D spheroids was confirmed by real-time RT-PCR (Fig. S4). Our results showed that CLCN3 shRNA stable cells have significantly decreased 3D spheroid proliferation compared with control shRNA stable cells in both SKBR3 and MDA-MB-453 cells (Fig. 5B, Fig. S5).

## Discussion

To understand the comprehensive role of miR-205-5p in breast cancer, we attempted to identify a novel target of miR-205-5p that may be involved in breast cancer progression and performed analyses focusing on CLCN3. On the basis of our observation that CLCN3 expression was increased in the ErbB2-overexpressing breast epithelial cells MCF10A-ErbB2, and ectopic transfection of the miR-205-5p precursor reduced the elevated CLCN3 expression levels, CLCN3 may prove to be a miR-205-5p target. Interestingly, we additionally found that miR-205-5p expression was significantly reduced in the ErbB2-overexpressing breast cancer cell lines stably expressing CLCN3 shRNA (Fig. S6). This may occur by negative feedback loop mechanisms between miRNA and its target genes (Herranz & Cohen, 2010; Liu, Duan & Duan, 2018). Interaction between CLCN3 and miR-205-5p was also verified by the significantly high enrichment of CLCN3 in miR-205-5p mimic transfected 293T cells via RIP analysis. These observations were further confirmed by reporter assays revealing that the miR-205-5p mimic significantly decreased the reporter activity of CLCN3-3′-UTR-wt. Additional observations that the miR-205-5p mimic did not show significant effects on the reporter activity of the complete mutant, CLCN3-3′-UTR-mut1, strongly support that CLCN3 is likely a novel potential target of miR-205-5p. The seed sequence, nucleotides 2 to 8 of the miRNA, has been recognized as a critical determinant of canonical miRNA–target interaction. Although there are imperfect seed matches between CLCN3 and miR-205-5p, recent reports revealed that imperfect seed matches could be compensated for by extensive pairing with the seed-distal 3′ end of the miRNA (Brancati & Grosshans, 2018; Broughton et al., 2016). According to our data showing that the miR-205-5p mimic also significantly decreased the reporter activity of the partial mutants, CLCN3-3′-UTR-mut2 and CLCN3-3′-UTR-mut3, it is suggested that the miR-205-5p and CLCN3 interaction needs both seed and seed-distal pairing.

CLCN3 is a member of the voltage-gated chloride channel family and functions as a Cl^−^/H^+^ transporter in intracellular membranes (Duran et al., 2010; Guzman et al., 2013). In addition, several studies have shown that CLCN3 is involved in cell proliferation, apoptosis, drug resistance, and invasion in many cancers (Lui et al., 2010; Su et al., 2013; Xu et al., 2010; Zhang et al., 2013). In cellular proliferation, CLCN3 plays an important role by controlling cell cycle progression (Wang et al., 2002; Xu et al., 2010). Knockdown of CLCN3 by siRNA reduces cells in S phase, while increasing those in G_0_/G_1_ phase, in rat basilar arterial smooth muscle cells and inhibits cellular proliferation by downregulating the expression of cyclin D1 and cyclin E in mouse mesenchymal stem cells (Tang et al., 2008; Tao et al., 2008). CLCN3 also accelerates the G_0_/G_1_ to S phase transition in the cell cycle by enhancing the phosphorylation of ERK1/2 and upregulating cyclin E and cyclin D1 in multiple myeloma cells (Du et al., 2018). Thus, there are several reports on the functions of CLCN3 in cancer. However, the roles of CLCN3 in breast cancer cell proliferation remain unclear. Our findings in this study indicate that CLCN3 promotes 3D spheroid proliferation in ErbB2-overexpressing breast epithelial and cancer cells. These findings should improve our understanding of the significance of CLCN3 in breast cancer cellular proliferation. In addition, we showed that miR-205-5p expression in exosomes isolated from MCF10-neo cells was higher than in exosomes isolated from MCF10-ErbB2 cells. Our data of exosome treatment experiments further indicate that exosomal miR-205-5p may be functionally transferred between breast epithelial cells and inhibit 3D spheroid proliferation by downregulating CLCN3. Although the inhibitory effect of exosomal miR-205-5p on CLCN3 expression was marginal, it was likely due to the amount of exosomal miR-205-5p transfer being considerably small in comparison with that of miR-205-5p precursor or mimic transfection. The important thing was that miR-205-5p could be successfully and functionally transferred by exosome treatment. We also found that expression of miR-200 family members in exosomes from MCF10A-neo cells was significantly higher than in exosomes from MCF10-ErbB2 cells, but the transfer of miR-200 family members didn’t occur under the same exosome treatment condition (Figs. S7A and S7B). Our observation suggests that exosome-mediated miR-205-5p transfer may contribute to the control of cell proliferation in physiological conditions, and exosomes may be utilized to deliver exogenous miR-205-5p.

## Conclusions

In conclusion, our novel findings demonstrated that CLCN3 is a likely target of miR-205-5p, and overexpression of ErbB2 induces CLCN3 expression by downregulating miR-205-5p in breast epithelial cells. In addition, CLCN3 promotes 3D spheroid proliferation in ErbB2-overexpressing breast epithelial and cancer cells. On the basis of these observations, CLCN3 may have the potential to be a novel therapeutic target for ErbB2-overexpressing breast cancers.

##  Supplemental Information

10.7717/peerj.7799/supp-1 Supplemental Information 1 Supplementary Material and Methods

10.7717/peerj.7799/supp-2 Figure S1 Detection of exosomal protein markers in exosome derived from MCF10A-neo and MCF10A-ErbB2 cells Western blot analysis of exosome isolated from MCF10A-neo and MCF10A-ErbB2 cells. The isolated exosome was mixed directly with Laemmli sample buffer and subjected to SDS-PAGE. Each sample represented exosomes isolated from equal number of cells.

10.7717/peerj.7799/supp-3 Figure S2 Expression of miR-205 in exosomes derived from MCF10A-neo and MCF10A-ErbB2 cells Real-time RT-PCR analysis for miR-205-5p using total RNA purified from exosome derived from MCF10A-neo and MCF10A-ErbB2 cells. Data were normalized to vehicle control and represented as the mean ± SEM of three independent experiments. * *p* < 0.01 by Student’s *t*-test compared with vehicle.

10.7717/peerj.7799/supp-4 Figure S3 Generation of CLCN3 shRNA stable cells Western blot analysis of SKBR3 and MDA-MB-453 cells stably expressing CLCN3 shRNA or control shRNA. β-actin was used as a control for loading. The numbers below the bands indicate fold change as compared to the corresponding control shRNA stable cells, upon normalization to β-actin.

10.7717/peerj.7799/supp-5 Figure S4 Expression of CLCN3 in 3D spheroids of CLCN3 shRNA stable cells Real-time RT-PCR analysis for CLCN3 using total RNA purified from 3D spheroids of SKBR3 and MDA-MB-453 cells stably expressing CLCN3 shRNA or control shRNA. Data were normalized to control shRNA stable cells and represented as the mean ± SEM of three independent experiments. * *p* < 0.01 by Student’s *t*-test compared with vehicle.

10.7717/peerj.7799/supp-6 Figure S5 3D spheroid proliferation assay CLCN3 shRNA or control shRNA stably expressing SKBR3 and MDA-MB-453 cells were applied for 3D spheroid proliferation assay for 6 days. Representative pictures of spheroid in each group are shown.

10.7717/peerj.7799/supp-7 Figure S6 Expression of miR-205-5p in CLCN3 shRNA stable cells Real-time RT-PCR analysis for miR-205-5p using total RNA purified from SKBR3 and MDA-MB-453 cells stably expressing CLCN3 shRNA or control shRNA. Data were normalized to control shRNA stable cells and represented as the mean ± SEM of three independent experiments. * *p* < 0.05 by Student’s *t*-test compared with vehicle.

10.7717/peerj.7799/supp-8 Figure S7 Expression of miR-200 family members (A) Real-time RT-PCR analysis for miR-200a-3p, miR-200b-3p, miR-200c-3p, miR-141-3p and miR-429 using total RNA purified from exosome derived from MCF10A-neo and MCF10A-ErbB2 cells. Data were normalized to vehicle control and represented as the mean ±SEM of three independent experiments. * *p* < 0.01 by Student’s *t*-test compared with vehicle. (B) Real-time RT-PCR analysis for miR-200a-3p, miR-200b-3p, miR-200c-3p, miR-141-3p and miR-429. MCF10A-ErbB2 cells were treated with MCF10A-neo-derived exosome for 24 h. Data were normalized to vehicle control and represented as the mean ± SEM of three independent experiments. MiR-429 expression was undetectable.

10.7717/peerj.7799/supp-9 Data S1 Raw data

10.7717/peerj.7799/supp-10 Supplemental Information 2 Full-length uncropped blots

## Additional Information and Declarations

### Data Availability

The following information was supplied regarding data availability: The raw measurements are available in the Supplemental Files.
