# Supplementary material for: MicroRNA-205-5p inhibits three-dimensional spheroid proliferation of ErbB2-overexpressing breast epithelial cells through direct targeting of CLCN3
Source: PeerJ. 2019 Oct 8;7:e7799. doi: 10.7717/peerj.7799 (PMC6788438; doi:10.7717/peerj.7799)
Supplement: Supplemental Information 1 [file peerj-07-7799-s001.docx]

**Supplementary Material and Methods**

The following antibodies were used for western blotting analysis: ab28736, a rabbit monoclonal antibody to CLCN3 (Abcam, Cambridge, UK); 4872, a rabbit monoclonal antibody to HSP70 (Cell Signaling Technology, Danvers, MA); EXOAB-CD63A-1, a rabbit polyclonal antibody to CD63 (System Biosciences, Palo Alto, CA); and MAB1501, a mouse monoclonal antibody to Actin (Millipore, Billerica, MA), as a protein loading control and horseradish peroxidase-conjugated goat antibodies to mouse and rabbit IgG (Santa Cruz Biotechnology).
