# Supplementary figures and images for: MicroRNA-205-5p inhibits three-dimensional spheroid proliferation of ErbB2-overexpressing breast epithelial cells through direct targeting of CLCN3
Source: PeerJ. 2019 Oct 8;7:e7799. doi: 10.7717/peerj.7799 (PMC6788438; doi:10.7717/peerj.7799)

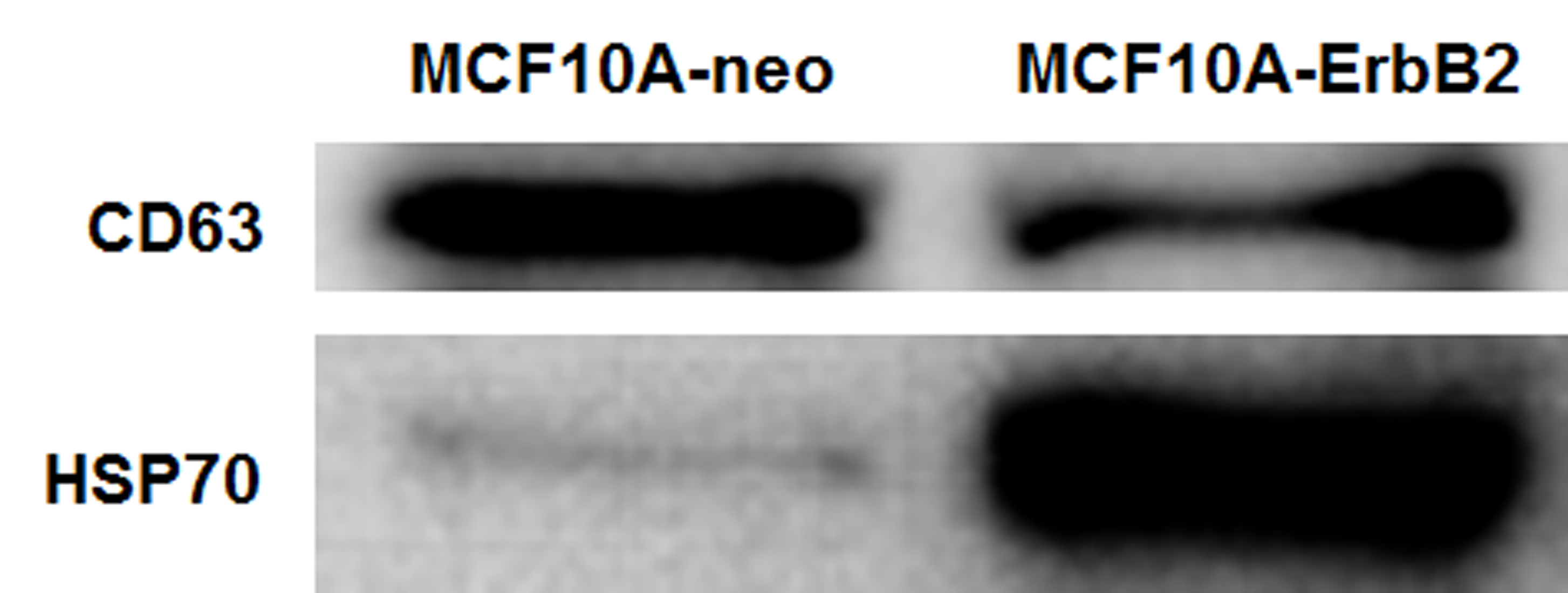

Supplement: Figure S1 — Western blot analysis of exosome isolated from MCF10A-neo and MCF10A-ErbB2 cells. The isolated exosome was mixed directly with Laemmli sample buffer and subjected to SDS-PAGE. Each sample represented exosomes isolated from equal number of cells. [file peerj-07-7799-s002.png]

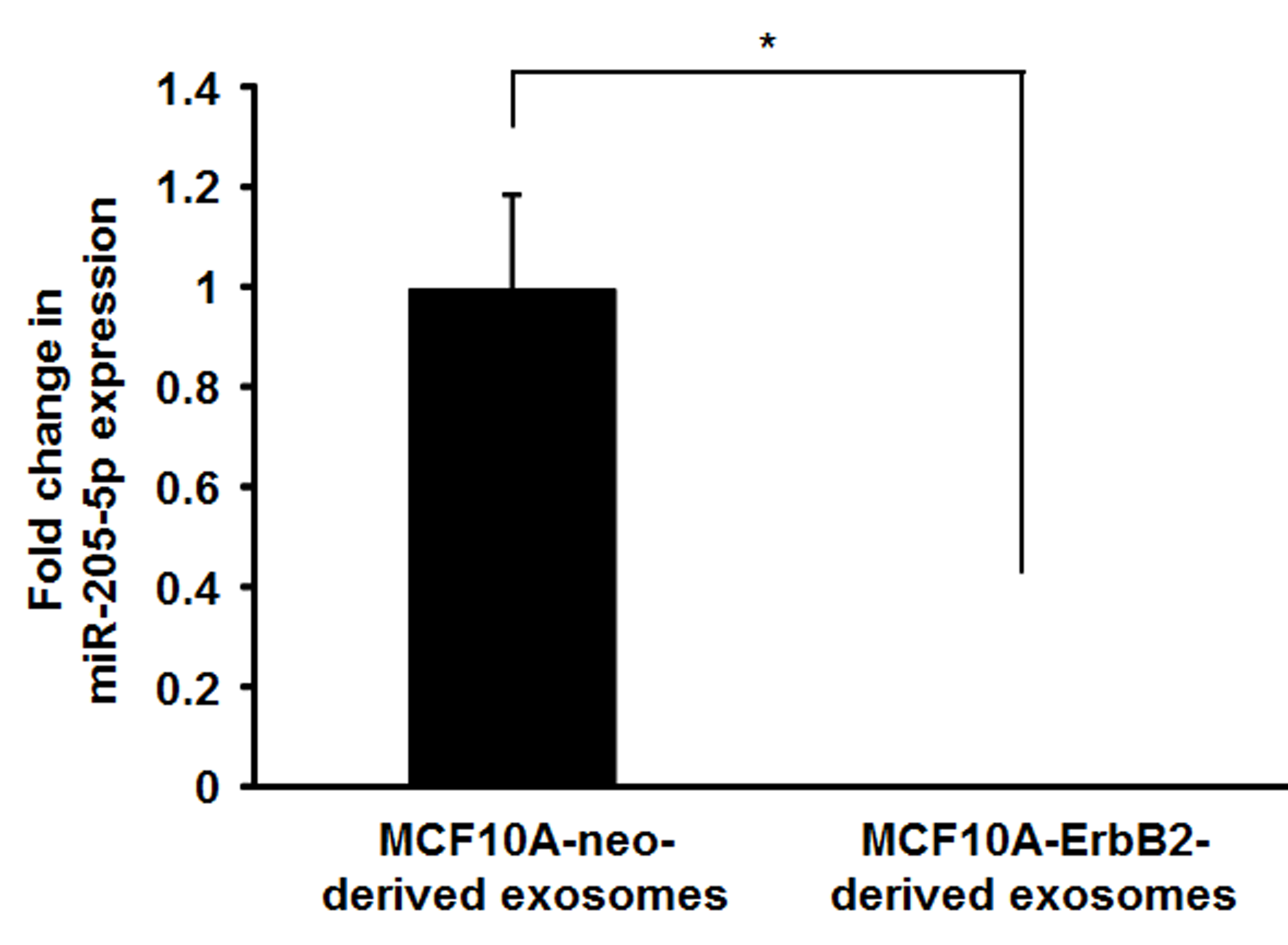

Supplement: Figure S2 — Real-time RT-PCR analysis for miR-205-5p using total RNA purified from exosome derived from MCF10A-neo and MCF10A-ErbB2 cells. Data were normalized to vehicle control and represented as the mean ± SEM of three independent experiments. * p < 0.01 by Student’s t-test compared with vehicle. [file peerj-07-7799-s003.png]

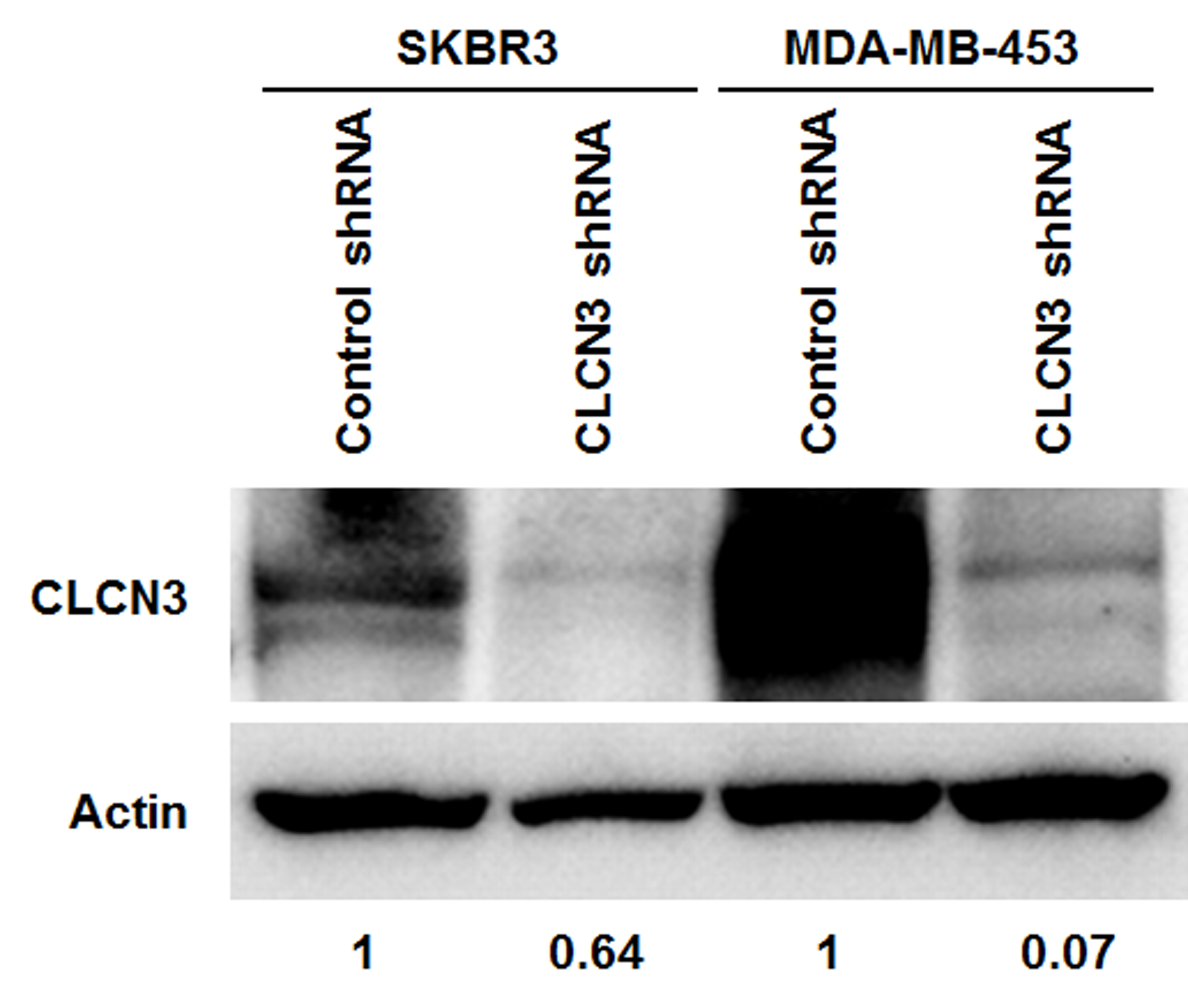

Supplement: Figure S3 — Western blot analysis of SKBR3 and MDA-MB-453 cells stably expressing CLCN3 shRNA or control shRNA. β-actin was used as a control for loading. The numbers below the bands indicate fold change as compared to the corresponding control shRNA stable cells, upon normalization to β-actin. [file peerj-07-7799-s004.png]

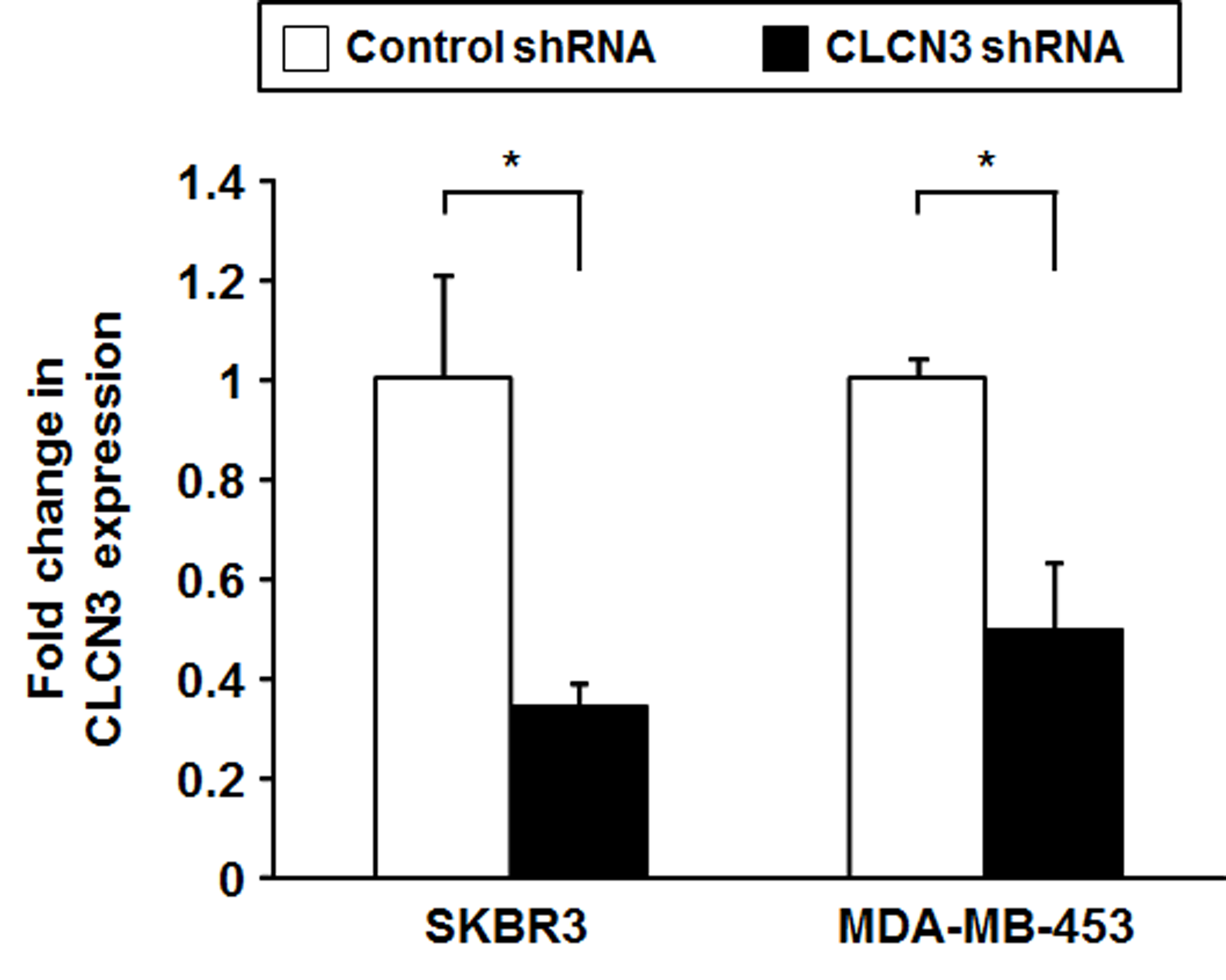

Supplement: Figure S4 — Real-time RT-PCR analysis for CLCN3 using total RNA purified from 3D spheroids of SKBR3 and MDA-MB-453 cells stably expressing CLCN3 shRNA or control shRNA. Data were normalized to control shRNA stable cells and represented as the mean ± SEM of three independent experiments. * p < 0.01 by Student’s t-test compared with vehicle. [file peerj-07-7799-s005.png]

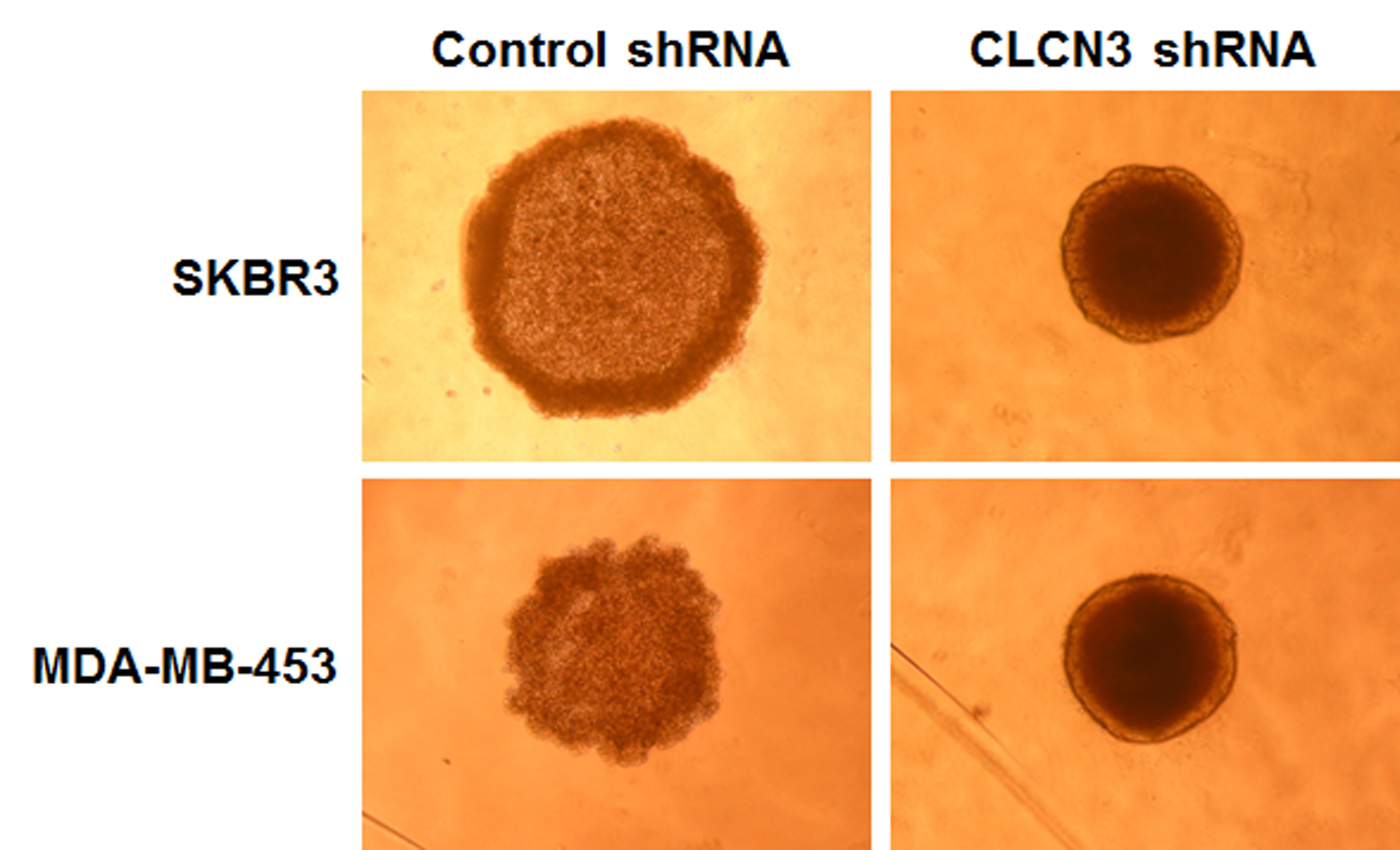

Supplement: Figure S5 — CLCN3 shRNA or control shRNA stably expressing SKBR3 and MDA-MB-453 cells were applied for 3D spheroid proliferation assay for 6 days. Representative pictures of spheroid in each group are shown. [file peerj-07-7799-s006.png]

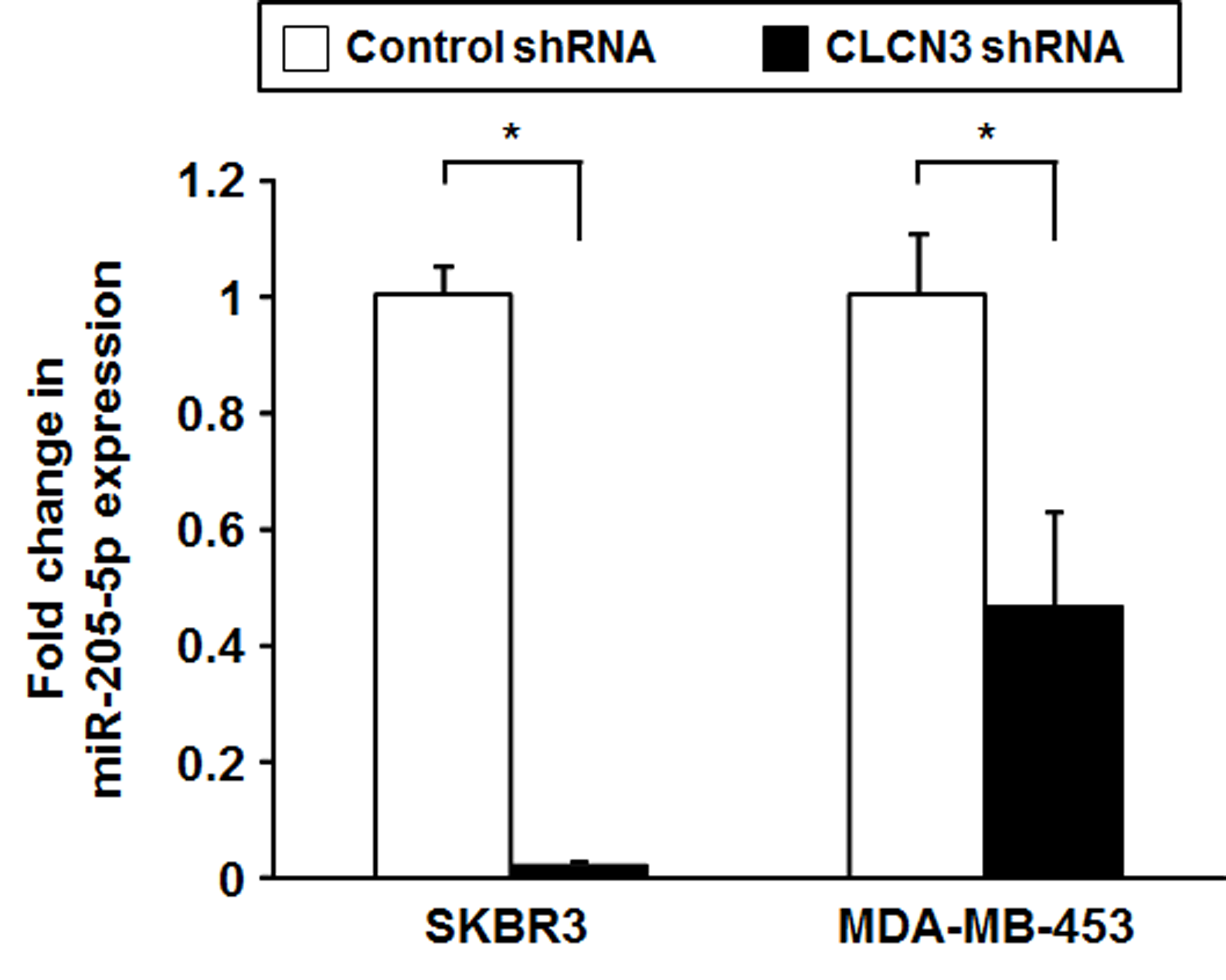

Supplement: Figure S6 — Real-time RT-PCR analysis for miR-205-5p using total RNA purified from SKBR3 and MDA-MB-453 cells stably expressing CLCN3 shRNA or control shRNA. Data were normalized to control shRNA stable cells and represented as the mean ± SEM of three independent experiments. * p < 0.05 by Student’s t-test compared with vehicle. [file peerj-07-7799-s007.png]

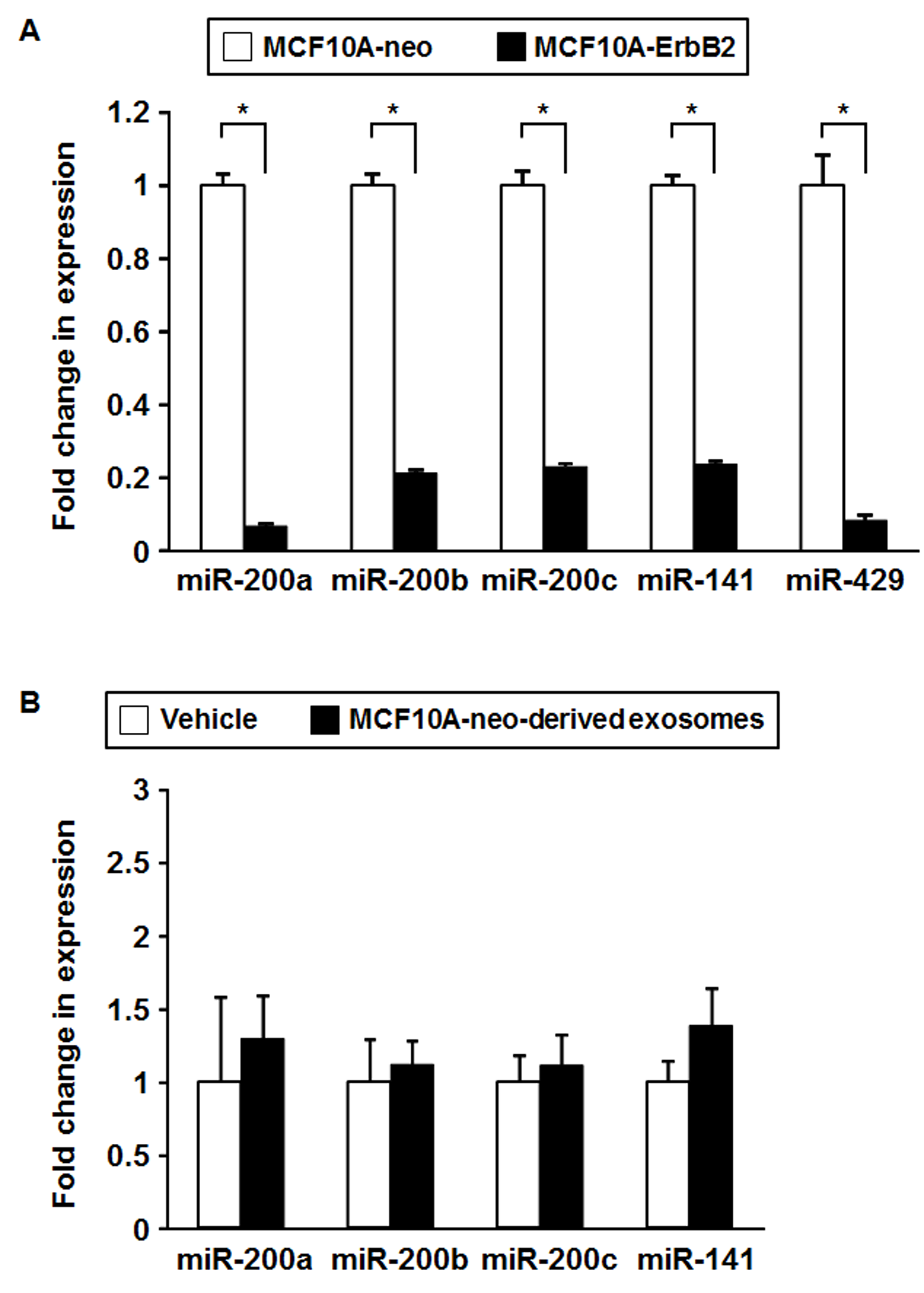

Supplement: Figure S7 — (A) Real-time RT-PCR analysis for miR-200a-3p, miR-200b-3p, miR-200c-3p, miR-141-3p and miR-429 using total RNA purified from exosome derived from MCF10A-neo and MCF10A-ErbB2 cells. Data were normalized to vehicle control and represented as the mean ±SEM of three independent experiments. * p < 0.01 by Student’s t-test compared with vehicle. (B) Real-time RT-PCR analysis for miR-200a-3p, miR-200b-3p, miR-200c-3p, miR-141-3p and miR-429. MCF10A-ErbB2 cells were treated with MCF10A-neo-derived exosome for 24 h. Data were normalized to vehicle control and represented as the mean ± SEM of three independent experiments. MiR-429 expression was undetectable. [file peerj-07-7799-s008.png]

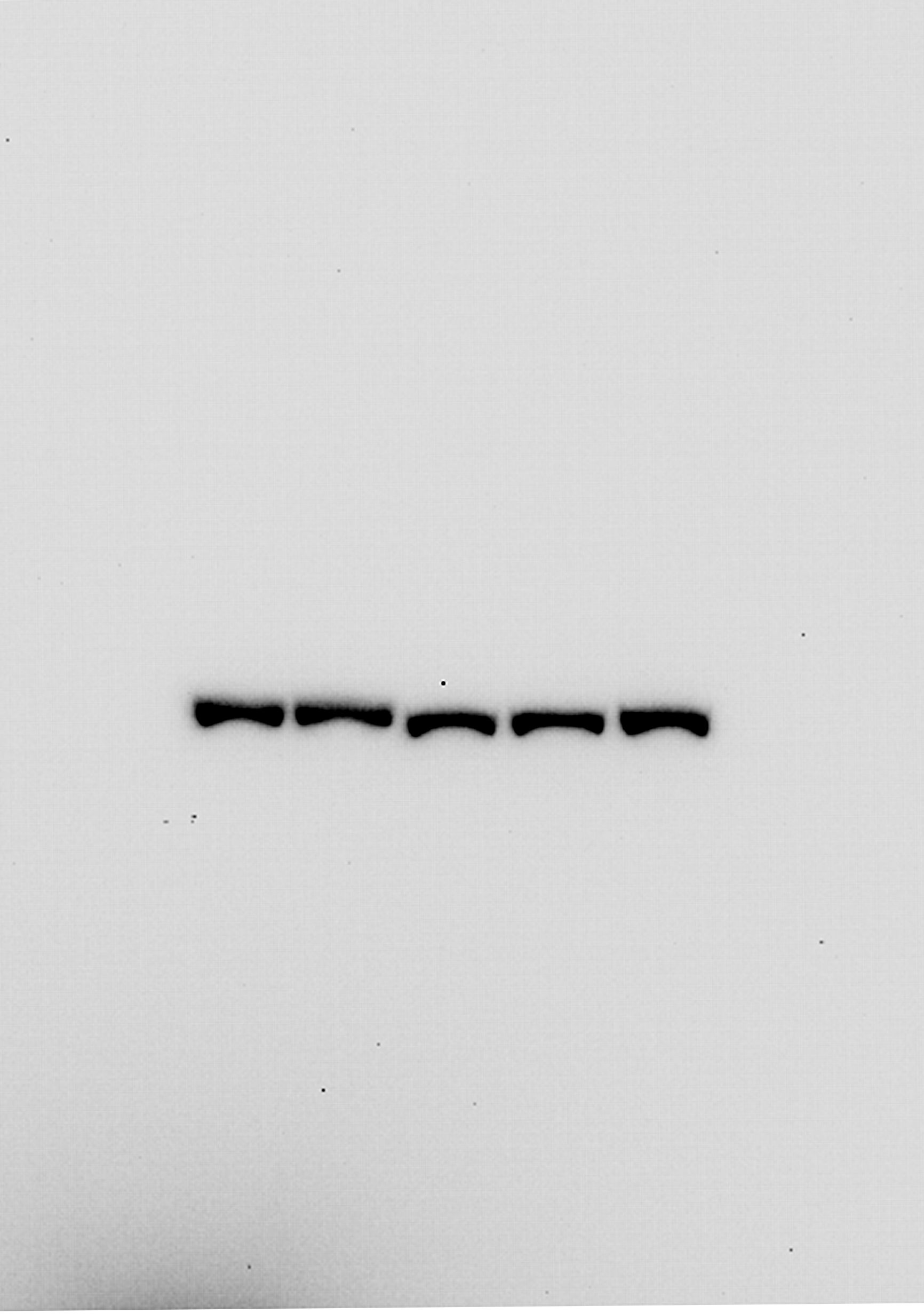

Supplement: Supplemental Information 2 [file peerj-07-7799-s010.zip › Uncut images/Actin uncut.tif]

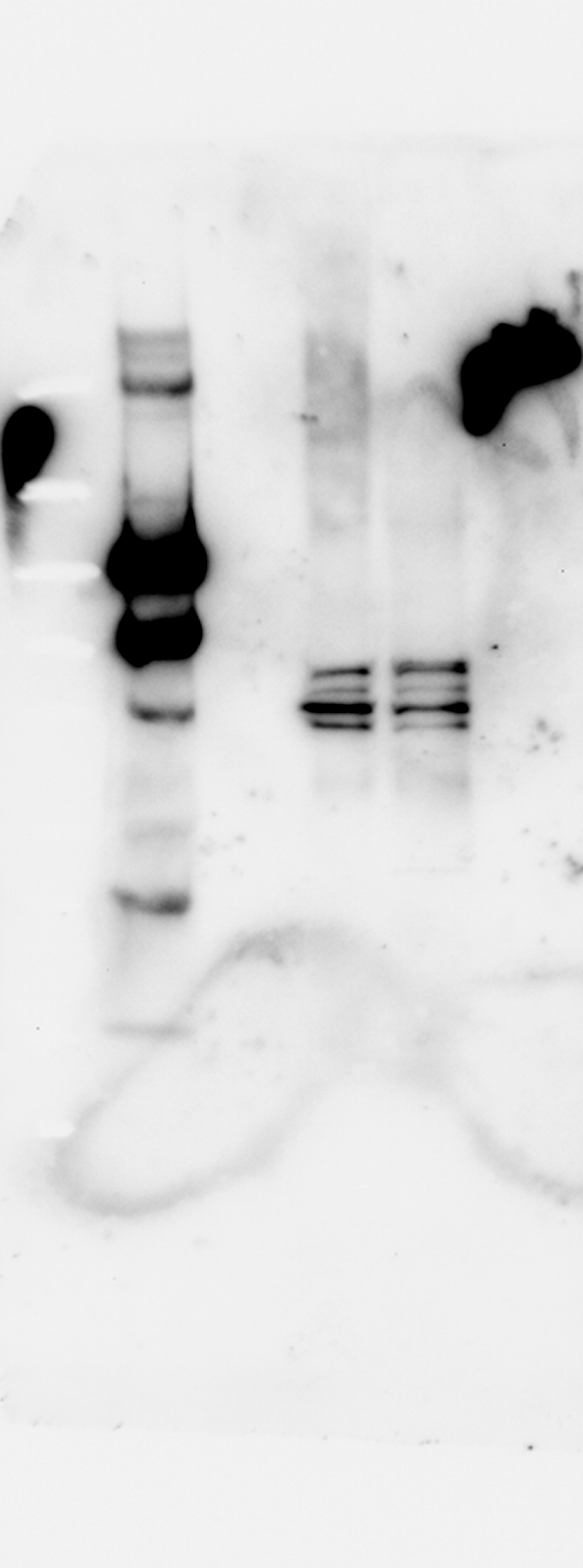

Supplement: Supplemental Information 2 [file peerj-07-7799-s010.zip › Uncut images/CD63 uncut.tif]

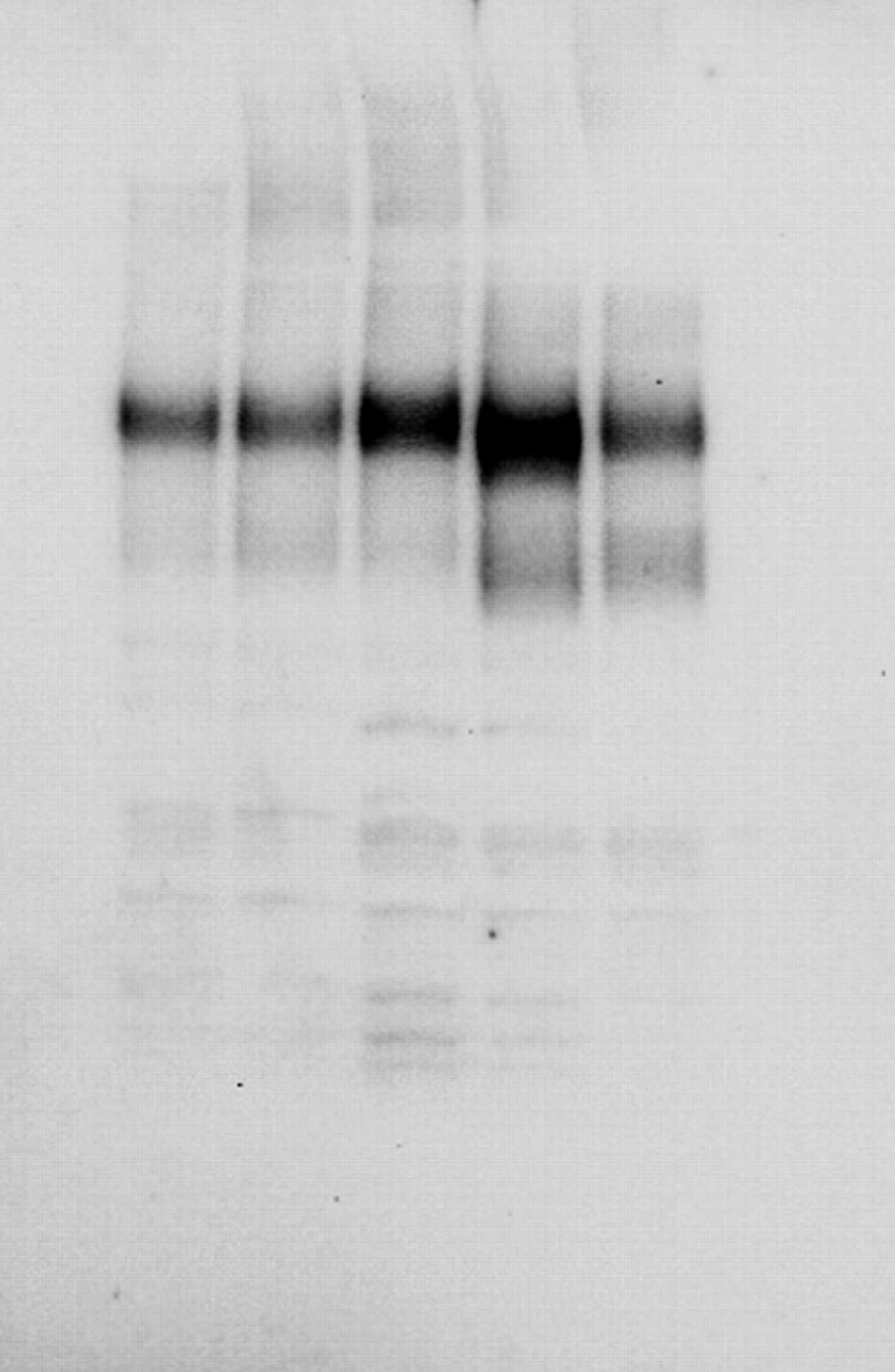

Supplement: Supplemental Information 2 [file peerj-07-7799-s010.zip › Uncut images/CLCN3 uncut.tif]

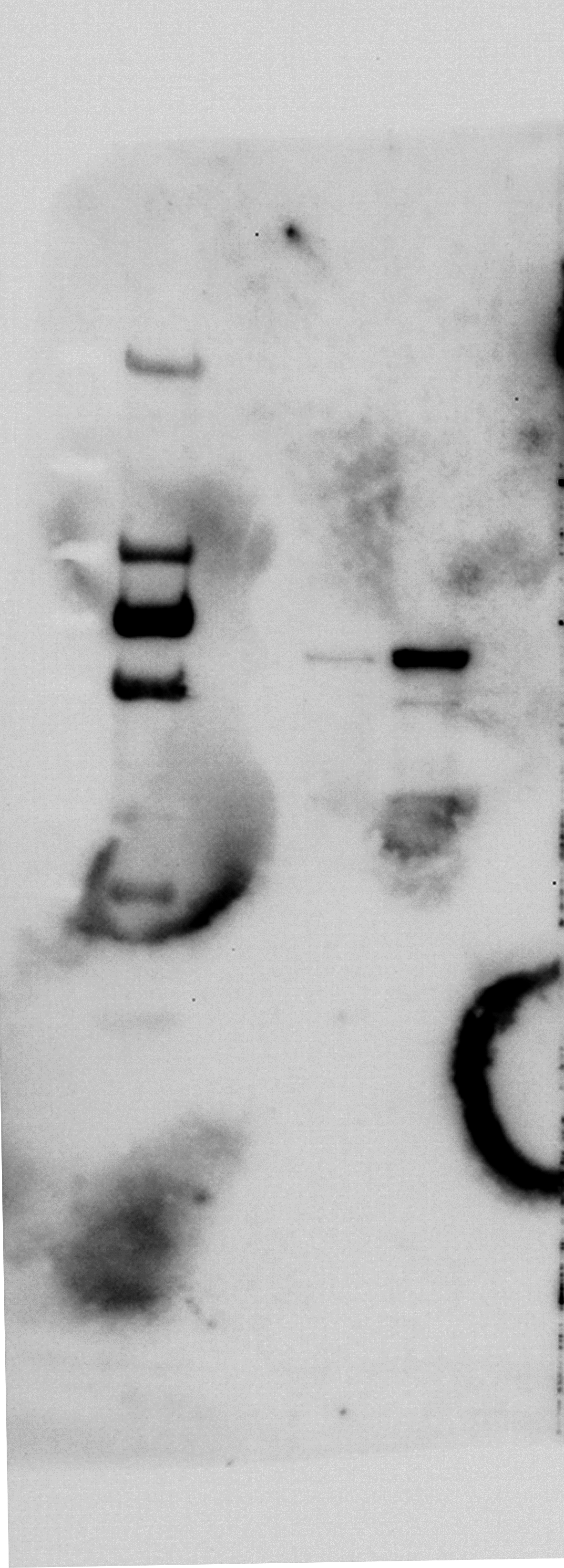

Supplement: Supplemental Information 2 [file peerj-07-7799-s010.zip › Uncut images/HSP70 uncut.tif]

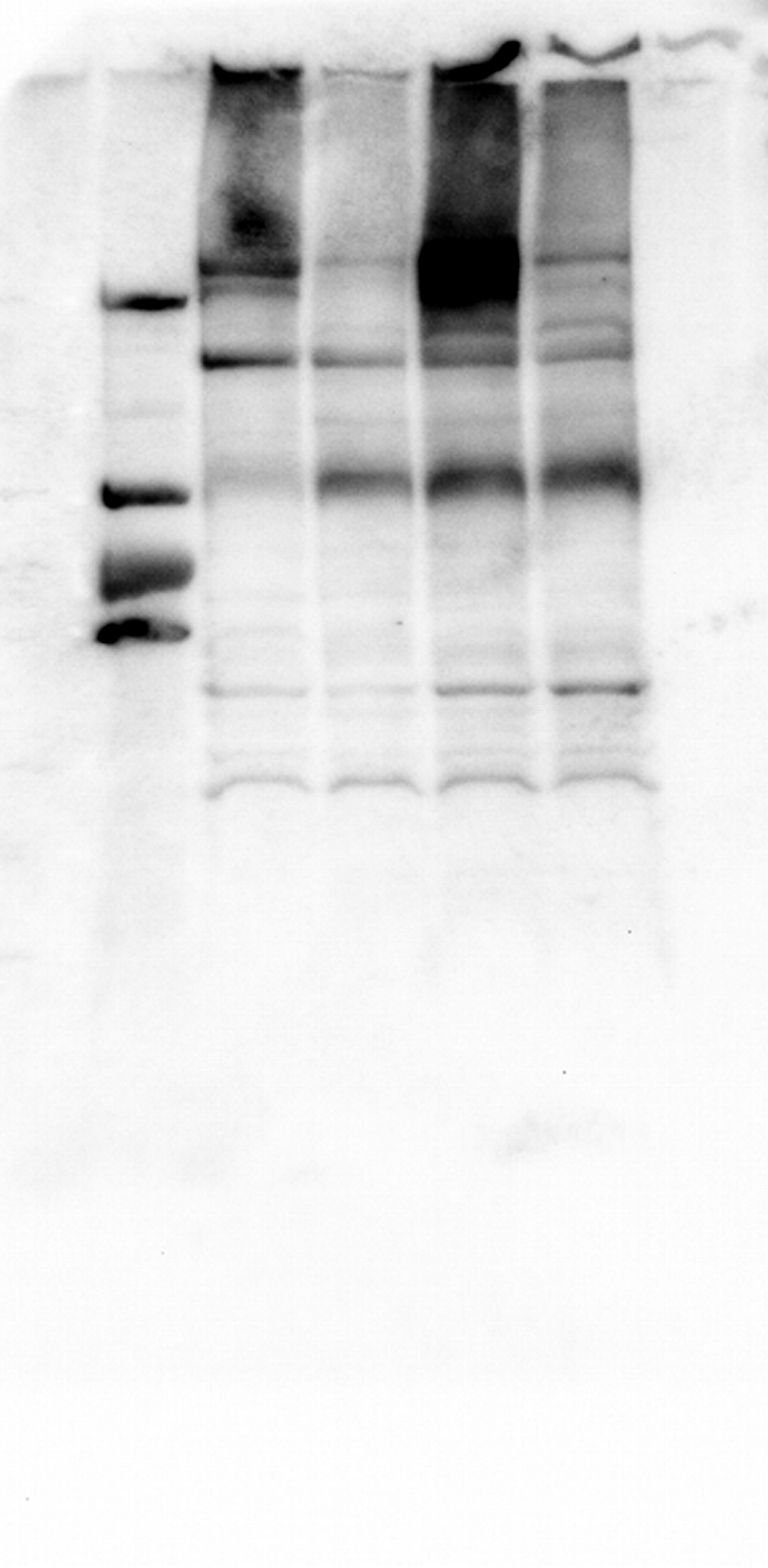

Supplement: Supplemental Information 2 [file peerj-07-7799-s010.zip › Uncut images/Stable cells CLCN3 uncut.tif]
